# Optimal Cerebral Perfusion Pressure Guided by Brain Oxygen Pressure Measurement

**DOI:** 10.3389/fneur.2021.732830

**Published:** 2021-10-28

**Authors:** Matyas Kovacs, Lorenzo Peluso, Hassane Njimi, Olivier De Witte, Elisa Gouvêa Bogossian, Armin Quispe Cornejo, Jacques Creteur, Sophie Schuind, Fabio Silvio Taccone

**Affiliations:** ^1^Department of Intensive Care, Hopital Erasme, Université Libre de Bruxelles, Brussels, Belgium; ^2^Department of Neurosurgery, Hopital Erasme, Université Libre de Bruxelles, Brussels, Belgium

**Keywords:** brain injury, brain oxygenation, optimal perfusion, individualized therapy, traumatic brain injury, intracranial hemorrhage, subarachnoid hemorrhage

## Abstract

**Background:** Although increasing cerebral perfusion pressure (CPP) is commonly accepted to improve brain tissue oxygen pressure (PbtO_2_), it remains unclear whether recommended CPP targets (i. e., >60 mmHg) would result in adequate brain oxygenation in brain injured patients. The aim of this study was to identify the target of CPP associated with normal brain oxygenation.

**Methods:** Prospectively collected data including patients suffering from acute brain injury and monitored with PbtO_2_, in whom daily CPP challenge using vasopressors was performed. Initial CPP target was >60 mmHg; norepinephrine infusion was modified to have an increase in CPP of at least 10 mmHg at two different steps above the baseline values. Whenever possible, the same CPP challenge was performed for the following days, for a maximum of 5 days. CPP “responders” were patients with a relative increase in PbtO_2_ from baseline values > 20%.

**Results:** A total of 53 patients were included. On the first day of assessment, CPP was progressively increased from 73 (70–76) to 83 (80–86), and 92 (90–96) mmHg, which resulted into a significant PbtO_2_ increase [from 20 (17–23) mmHg to 22 (20–24) mmHg and 24 (22–26) mmHg, respectively; *p* < 0.001]. Median CPP value corresponding to PbtO_2_ values > 20 mmHg was 79 (74–87) mmHg, with 2 (4%) patients who never achieved such target. Similar results of CPP targets were observed the following days. A total of 25 (47%) were PbtO_2_ responders during the CPP challenge on day 1, in particular if low PbtO_2_ was observed at baseline.

**Conclusions:** PbtO_2_ monitoring can be an effective way to individualize CPP values to avoid tissue hypoxia. Low PbtO_2_ values at baseline can identify the responders to the CPP challenge.

## Introduction

Monitoring of patients with critical illness has expanded significantly over the past several decades. As for hemodynamics, respiratory or renal functions, several studies have underlined the importance of monitoring brain function, not only in patients with a primary brain injury [i.e., traumatic brain injury (TBI), subarachnoid hemorrhage (SAH), post-anoxic brain injury], but also in those with a systemic disease, such as sepsis, acute pancreatitis, or cardiogenic shock, who may present different alterations of cerebral function and potentially may develop secondary brain injuries (1), which can impact on long-term functional and cognitive recovery (2).

In patients suffering from TBI, the combination of intracranial pressure (ICP) and cerebral perfusion pressure (CPP)-guided therapy is recommended for the current management of these patients (3); similarly, ICP and CPP are the cornerstone of management of severe non-traumatic brain injury patients, although the evidence suggesting that such approach might impact on long-term outcomes remain limited (4). Importantly, increased ICP could be a late and insensitive indicator of some secondary brain injuries (5, 6); in particular, the occurrence of tissue hypoxia may occur even in the absence of intracranial hypertension and, when untreated or refractory to therapeutic interventions, is associated with an increased risk of poor neurological outcome in brain injured patients (7, 8). As such, rather than protocolizing therapies to target fixed ICP and CPP values for all patients, independently from the underlying brain injury, comorbid diseases, and the severity of brain edema, the implementation of brain tissue oxygen pressure (PbtO_2_) monitoring could be helpful to individualize ICP and CPP values based on cerebral oxygenation.

Although higher than recommended CPP targets (i.e., >60 mmHg) have been associated with adequate PbtO_2_ values in heterogeneous populations of brain injured patients, few prospective studies have evaluated the effects of increasing CPP using vasopressors on PbtO_2_ in such patients. In one small study (*n* = 11) on TBI patients, increasing CPP from 70 to 90 mmHg with norepinephrine resulted in a significant increase in PbtO_2_ (from 17 ± 8 to 22 ± mmHg), despite brain metabolism was only marginally affected (8). In another study including 14 TBI patients, increasing CPP resulted also in a significant increase in PbtO_2_, although this was measured after a challenge of inspired oxygen at 100% on the ventilator (9). However, all these studies did not specifically investigate the proportion of patients requiring higher than recommended CPP to obtain adequate PbtO_2_ values, neither whether this CPP target would change along the hospital stay.

The aim of this study was therefore to evaluate which level of CPP corresponds to an adequate PbtO_2_ in an heterogeneous population of brain injured patients. Secondary aims were to assess: (a) whether this level of CPP change over time and (b) the characteristics of patients requiring higher than recommended CPP targets to have optimal brain oxygenation values.

## Methods

### Study Population

This was an analysis of prospectively collected data including all adult (>18 years of age) patients with an acute primary brain injury admitted to the ICU of Erasme Hospital, Brussels, Belgium, between January 2016 and December 2020. Eligible patients were those: (a) having a PbtO_2_ monitoring catheter, which was inserted according to the decision of senior ICU physician and an experienced neurosurgeon; (b) receiving vasopressors to target a CPP of at least 60–70 mmHg; (c) who underwent daily CPP challenge as part of routine patient management (see below). Data for all measurements were recorded into the patient management data system (PDMS, Picis Critical Care Manager; Picis Inc., Wakefield, MA, USA). Exclusion criteria were a malfunctioning PbtO_2_ catheter, the absence of vasopressor therapy, elevated ICP (>25 mmHg) refractory to different interventions (i.e., sedation, osmotic therapy, and hyperventilation), clinical contraindication to increased CPP (i.e., frequent arrhythmias or acute heart failure). The study was approved by the ethical committee of the Erasme Hospital (Comité d'Ethique Hospitalo-Facultaire Erasme – ULB; P2021/038), which waived the need of informed consent given the observational design of the study analyzing recorded data into the PDMS.

### Patients' Management and CPP Challenge

Patients were managed according to local protocols, based on international recommendations (3, 4, 10, 11). A triple lumen bolt allowing the insertion of a PbtO_2_ probe (IM3.ST_EU; Integra LifeSciences Corporation, Plainsboro, NJ, USA), alone or in association with an eight-contact depth EEG electrode and a microdialysis catheter, was placed in the operating room by a neurosurgeon in patients with TBI, subarachnoid hemorrhage (SAH), or intracranial hemorrhage (ICH), who had indications for ICP monitoring (i.e., abnormal CT-scan findings and a Glasgow Coma Score on admission <9). The bolt was positioned in the normal-appearing brain area of the injured side or, in case of aneurysmal SAH, on either the ipsilateral side of the aneurysm (i.e., anterior circulation) or on the right side (i.e., no aneurysm identified or aneurysm located in the posterior circulation). Other continuously monitored variables included electrocardiogram, mean arterial pressure (MAP), peripheral oxygen saturation, end-tidal carbon dioxide and body temperature (i.e., with urinary or esophageal probes), as a routine approach in all severe brain injured patients.

For patients requiring vasopressors, initial CPP and PbtO_2_ targets are >60 mmHg and >20 mmHg, respectively. Cerebral perfusion pressure (CPP) was calculated as the difference between MAP and ICP; MAP was zeroed at the level of the left atrium (i.e., with patient at 30° recumbent position). After the initial daily assessment of the patient, which can also include the evaluation of pupillary function using an automated pupillometry (NeurOptics NPi-200; Neuroptics, Irvine, CA, USA) to calculate the Neurological Pupil Index (NPi), or brain flow velocities with Transcranial Doppler (TCD) to measure mean flow velocities (mFV) and the pulsatility index (PI) (12), CPP was set around 60–70 mmHg (i.e., if not already within these ranges) and norepinephrine infusion was modified to have an increase in CPP of at least 10 mmHg at two different steps above the baseline values. At each CPP level (i.e., stabilized for at least 5 min), PbtO_2_, ICP, NPi, mFV, and PI were collected and results used to adjust vasopressor infusion daily in order to define CPP goals according to PbtO_2_ values (i.e., target ≥ 20 mmHg) and/or guide further interventions. This CPP challenge was part of the routine management of patients, as it had a short duration (i.e., 10–15 min) and was performed by an experienced intensivist (FST), whenever possible. Apart from the CPP intervention, all other relevant physiological variables were kept stable. Also, the same CPP challenge was performed for the following days, always initiating on a CPP around 60–70 mmHg and testing two additional steps. A maximum of 5 days of testing was considered (i.e., either brain oxygenation was normalized and the catheter removed or increased ICP or persistent brain hypoxia would prevent further testing); CPP “responders” were defined as those patients with a relative increase in PbtO_2_ from baseline values > 20%.

### Data Collection

For all patients, demographics, comorbid diseases, reasons for ICU admission as well as ICU length of stay and hospital mortality were collected. The severity of disease scores [i.e., Glasgow Coma Scale on admission, World Federation of Neurological Surgeons (WFNS) score in SAH patients; Marshall (13) and modified Fisher scores (14, 15) for cerebral CT-scan in TBI or SAH patients, respectively; and location and volume of ICH] were collected. The use of different therapies (i.e., mechanical ventilation, sedative, analgesic, vasopressor, inotrope, antiepileptic, barbituric, and/or osmotic drugs), as well as different interventions (i.e., ICP monitoring, hypothermia, hypocapnia, and decompressive craniotomy) was collected. Intracranial hypertension was defined by the observation of at least one ICP value above 20 mmHg for at least 5 min at any time. Brain tissue hypoxia was defined by a PbtO_2_ below 20 mmHg.

Neurological outcome at hospital discharge was assessed using the Glasgow Outcome Scale (GOS) (15); favorable neurological outcome (FO) was considered as a GOS 4–5, while unfavorable outcome (UO) as GOS 1–3.

### Study Outcomes

The primary outcome of the study was to evaluate which level of CPP corresponds to a PbtO_2_ ≥ 20 mmHg. Secondary outcomes included: (a) proportion and characteristics of PbtO_2_ responders; (b) comparison of PbtO_2_ changes according to the presence of baseline tissue hypoxia; (c) comparison of PbtO_2_ changes over time; (d) association of PbtO_2_ changes with NPi, mFV, and/or PI changes; (e) differences in PbtO_2_ changes during CPP challenges according to neurological outcome.

### Statistical Analysis

Data were analyzed using R statistical software version 4.0.3 (R Foundation for Statistical Computing), Prism (GraphPad Software Inc.), and IBM SPSS Statistics for Macintosh 27 (Armonk, NY, USA). Categorical variables were expressed as count (percentage) and continuous variables as mean ± standard deviation (SD) or median (25th−75th percentiles). The Kolmogorov-Smirnov test was used, and histograms and normal-quantile plots were examined to verify the normality of distribution of continuous variables. Differences between groups were assessed using the chi-square test or Fisher's exact test for categorical variables and Student's *t*-test, or Mann–Whitney *U*-test for continuous variables, as appropriate. Mixed model procedure with restricted maximum likelihood (REML) estimation and “unstructured” covariance structure was used to examine the differences in PbtO_2_ changes during CPP challenge over different days of assessment. All tests are two tailed and the statistical significance was set at the 5% level.

## Results

### Study Population

Over a total of 162 patients, 109 were excluded (i.e., *n* = 49, not on vasopressors during the PbtO_2_ monitoring period; *n* = 29, refractory intracranial hypertension; *n* = 31, measurements not performed) and 53 were eventually included into the final analysis. No significant differences were observed between included and excluded patients (Supplementary Table 1), except for a shorter ICU length of stay and higher mortality for the excluded group. Characteristics of the study population are shown in Table 1; the most frequent brain injury was SAH (*n* = 29, 55%). Hospital mortality occurred in 18 (34%) of patients, while 33 (62%) presented with UO.

**Table 1:** Characteristics of study population, according to neurological outcome (UO, unfavorable; FO, favorable).

	**Overall (*n* = 53)**	**UO (*n* = 33)**	**FO (*n* = 20)**	***p*-value**
**Demographics**				
Age, years	50 (40–58)	51 (44–58)	47 (38–57)	0.45
Male gender, *n* (%)	31 (58)	19 (58)	12 (60)	1.00
**Comorbidities**				
Hypertension, *n* (%)	14 (26)	10 (30)	4 (20)	0.53
Heart disease, *n* (%)	2 (4)	0	2 (10)	0.14
Alcohol, *n* (%)	12 (23)	7 (21)	5 (25)	0.75
Smoking, *n* (%)	12 (23)	6 (18)	6 (30)	0.34
Diabetes, *n* (%)	7 (13)	5 (15)	2 (10)	0.70
Previous neurological disease, *n* (%)	2 (4)	1 (3)	1 (5)	1.00
CKD, *n* (%)	0	0	0	
COPD, *n* (%)	4 (8)	3 (9)	1 (5)	1.00
Immunosuppression, *n* (%)	0	0	0	–
Cancer, *n* (%)	3 (6)	3 (9)	0	0.28
Liver cirrhosis, *n* (%)	1 (2)	1 (3)	0	1.00
**On admission**				
Glasgow coma scale	7 (3–10)	5 (3–9)	7 (5–13)	0.14
**Type of disease**, ***n*** **(%)**				0.25
*TBI*	20 (38)	10 (30)	10 (50)	
*SAH*	29 (55)	21 (64)	8 (40)	
*ICH*	4 (8)	2 (6)	2 (10)	
**During ICU stay**				
Vasopressors, *n* (%)	53 (100)	33 (100)	20 (100)	–
Mechanical ventilation, *n* (%)	53 (100)	33 (100)	20 (100)	–
RRT, *n* (%)	1 (2)	1 (3)	0	1.00
Osmotic therapy, *n* (%)	45 (85)	29 (88)	16 (80)	0.46
Decompressive craniectomy, *n* (%)	10 (19)	5 (15)	5 (25)	0.48
Hypothermia, *n* (%)	10 (19)	9 (27)	1 (5)	0.07
Anti-epileptics, *n* (%)	48 (91)	29 (88)	19 (95)	0.64
Barbiturates, *n* (%)	21 (40)	16 (48)	5 (25)	0.15
Intracranial hypertension, *n* (%)	36 (68)	26 (79)	10 (50)	0.04
Seizures, *n* (%)	9 (17)	5 (15)	4 (20)	0.72
**On the first day of assessment**				
Body temperature, °C	37 (36.8–37.3)	37 (36.8–37.3)	36.9 (36.7–37.3)	0.72
PaCO_2_, mmHg	37 (36–39)	37 (36–39)	37 (36–38)	0.95
PaO_2_, mmHg	102 (98–110)	100 (98–108)	104 (99–111)	0.38
Lactate, mmol/L	1 (0.8–1.2)	1 (0.8–1.2)	1.0 (0.8–1.1)	0.25
Hemoglobin, g/dl	11.3 (10.8–12.2)	11.2 (10.8–12.1)	11.4 (10.9–12.2)	0.88
FVm MCA, cm/sec	55.3 (48.5–65)	51.7 (47.5–67.3)	58.0 (54.7–63.2)	0.46
Pulsatility Index	0.90 (0.73–1.05)	0.93 (0.73–1.08)	0.85 (0.73–1.04)	0.77
Mean NPi	4.6 (4.03–4.66)	4.3 (3.2–4.7)	4.6 (4.3–4.6)	0.82
iPbtO_2_, mmHg	20 (17–23)	19 (16–23)	21 (18–24)	0.34
Brain hypoxia, *n* (%)	26 (49)	18 (55)	8 (40)	0.40
**Outcomes**				
ICU stay, days	21 (11–26)	17 (11–25)	24 (14–28)	0.15
Hospital mortality, *n* (%)	18 (34)	18 (55)	0	<0.01

*CKD, chronic kidney disease; COPD, chronic obstructive pulmonary disease; TBI, traumatic brain injury; SAH, subarachnoid hemorrhage; ICH, intracerebral hemorrhage; RRT, renal replacement therapy; PaCO_2_, partial arterial CO_2_ pressure; PaO_2_, partial arterial oxygen pressure; mFV-MCA, mean MCA flow velocity; NPI, neurological pupil index; iPbtO_2_, brain tissue oxygenation at baseline; ICU, intensive care unit*.

### CPP Challenge and PbtO_2_

**Figure 1:**
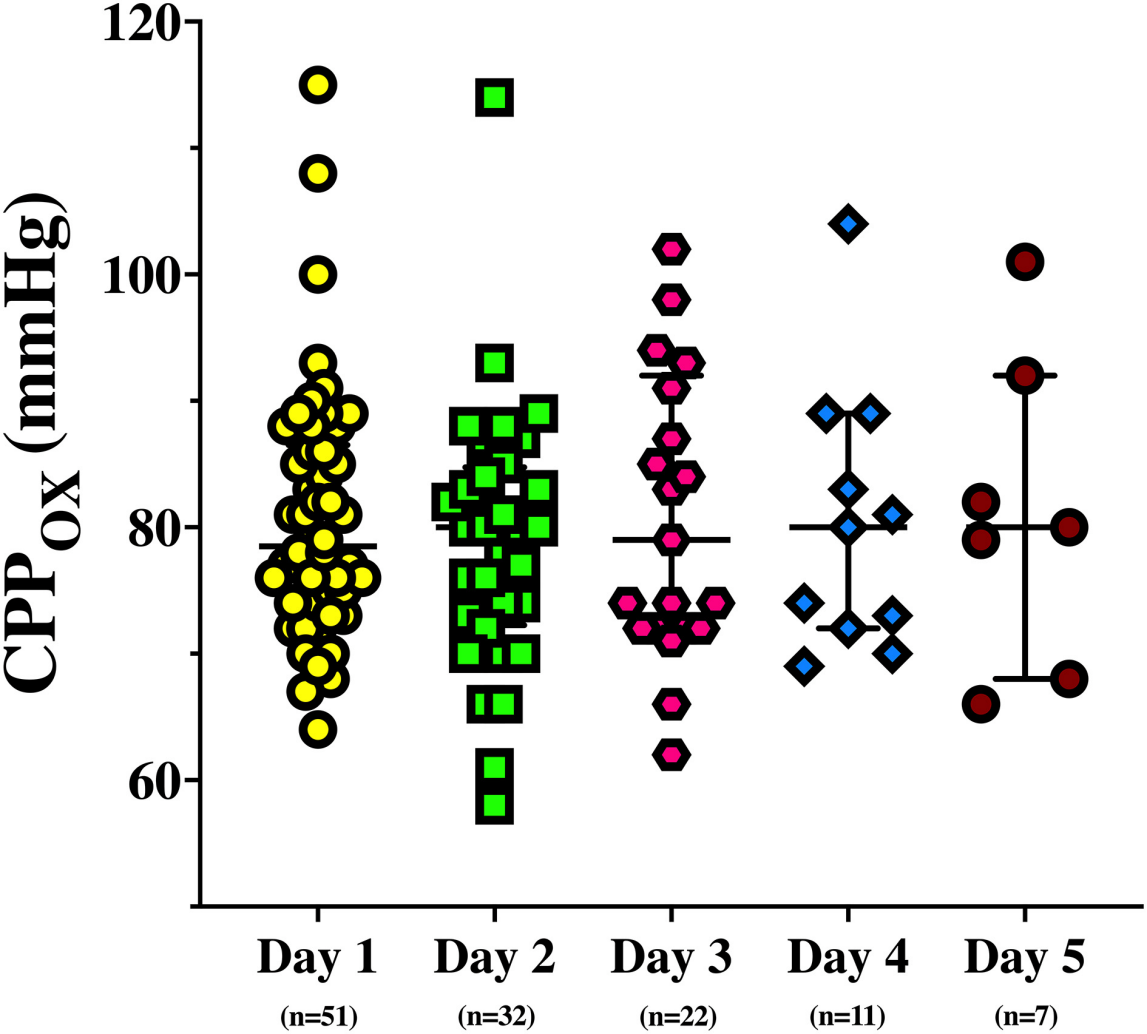
Cerebral perfusion pressure corresponding to a PbtO_2_ > 20 mmHg (CPP_OX_).

On the first day of assessment, baseline PbtO_2_ and CPP were 20 (16–21) mmHg and 73 (70–76) mmHg, respectively; brain hypoxia at baseline was observed in 26 (49%) of patients (Tables 1, 2). CPP was progressively increased to 83 (80–86) mmHg and 92 (90–96) mmHg, which resulted into a significant PbtO_2_ increase [to 22 (19–22) mmHg and 24 (20–24) mmHg, respectively; *p* < 0.001; Table 2]. During CPP increase, ICP also significantly decreased from baseline values. Median CPP value corresponding to PbtO_2_ values ≥ 20 mmHg on day 1 was 79 (74–87) mmHg, with 2 (4%) patients who never achieved such target; similar CPP values corresponding to PbtO_2_ values >20 mmHg were observed in traumatic (*n* = 38) and non-traumatic brain injury [79 (75–86) mmHg vs. 77 (72–88) mmHg; *p* = 0.52]. CPP challenges were repeated in 34 patients on day 2, 23 on day 3, 11 on day 4, and 7 on day 5; reasons for not performing CPP challenges the following days were mainly increased ICP (*n* = 4), the need for CPP > 90 mmHg to keep stable cerebral oxygenation (*n* = 5), or absence of operator and discontinuation of vasopressor therapy in others. Two patients on day 1 and 1 patient on day 3 never achieved the PbtO_2_ target. Similar values of CPP corresponding to PbtO_2_ values ≥ 20 mmHg were observed in the following days [80 (73–87) mmHg on day 2; 79 (72–92) mmHg on day 3; 80 (72–89) mmHg on day 4; 80 (68–92) mmHg on day 5; Figure 1]. No significant complications (i.e., increase in ICP, arrhythmias) during CPP challenge were reported.

**Table 2:** Changes in study variables on the first day of assessment, according to different steps of mean arterial pressure (MAP) and neurological outcome (UO, unfavorable; FO, favorable).

		**Step 1**	**Step 2**	**Step 3**	***p*-value**
All (*n* = 53)	MAP, mmHg	83 (82–89)	95 (91–99)	105 (100–109)	<0.01
	ICP, mmHg	12 (8–14)	12 (9–15)	11 (9–15)	<0.01
	CPP, mmHg	73 (70–76)	83 (80–86)	92 (90–96)	<0.01
	PbtO_2_, mmHg	20 (17–23)	22 (20–24)	24 (22–26)	<0.01
UO (*n* = 33)	MAP, mmHg	83 (81–87)	93 (91–96)	103 (100–110)	<0.01
	ICP, mmHg	10 (6–14)	11 (7–14)	11 (9–15)	<0.01
	CPP, mmHg	73 (70–75)	82 (79–85)	91 (89–96)	<0.01
	PbtO_2_, mmHg	19 (16–23)	22 (19–24)	24 (22–26)	<0.01
FO (*n* = 20)	MAP, mmHg	86 (82–90)	97 (94–100)	107 (102–110)	<0.01
	ICP, mmHg	13 (10–17)	12 (10–17)	13 (10–17)	0.42
	CPP, mmHg	74 (70–77)	85 (81–88)	93 (91–96)	<0.01
	PbtO_2_, mmHg	21 (18–24)	22 (21–25)	24 (23–28)	<0.01

*UO, unfavorable outcome; FO, favorable outcome; MAP, mean arterial pressure; ICP, intracranial pressure; CPP, cerebral perfusion pressure; PbtO_2_, brain tissue oxygenation*.

### Secondary Outcomes

**Figure 2:**
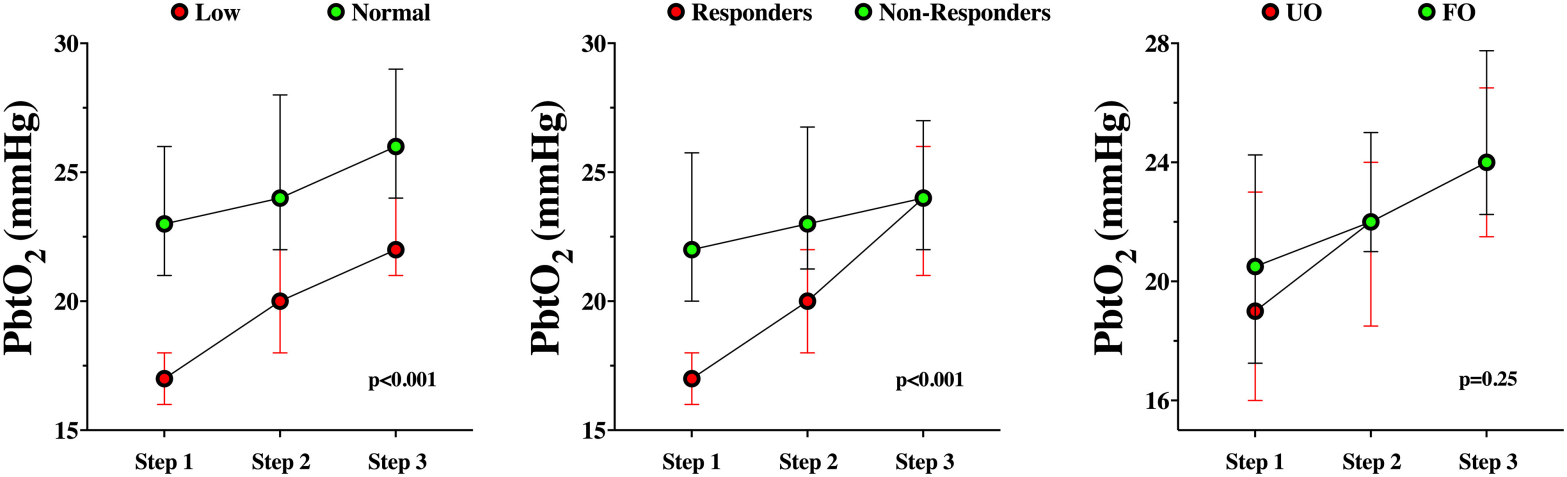
PbtO_2_ changes during the CPP challenge in responders and non-responders **(left panel)**, in baseline normal PbtO_2_ values or tissue hypoxia **(middle panel)** or according to the neurological outcome (UO, unfavorable; FO, favorable – **right panel**).

A total of 25 (47%) were PbtO_2_ responders during the CPP challenge on day 1. PbtO_2_ responders had similar characteristics to non-responders, including the presence of TBI, with the exception a lower PbtO_2_ at baseline (Table 3). Changes in PbtO_2_ on the first day of assessment were significantly higher over time in responders than in non-responders (Table 4; Figure 2).

**Table 3:** Characteristics of population according to PbtO_2_ responder or non-responder to CPP challenge.

	**Responder (*n* = 25)**	**Non responder(*n* = 28)**	***p*-value**
**Demographics**			
Age, years	52 (41–59)	50 (40–57)	0.57
Male gender, *n* (%)	11 (44)	20 (71)	0.06
**Comorbidities**			
Hypertension, *n* (%)	9 (36)	5 (18)	0.21
Heart disease, *n* (%)	0	2 (7)	0.49
Alcohol, *n* (%)	6 (24)	6 (21)	1.00
Smoking, *n* (%)	8 (32)	4 (14)	0.19
Diabetes, *n* (%)	2 (8)	5 (18)	0.43
Previous neurological disease, *n* (%)	0	2 (7)	0.49
CKD, *n* (%)	0	0	–
COPD, *n* (%)	1 (4)	3 (11)	0.61
Immunosuppression, *n* (%)	0	0	–
Cancer, *n* (%)	1 (4)	2 (7)	1.00
Liver Cirrhosis, *n* (%)	1 (4)	0	0.47
**On admission**			
Glasgow coma scale	6 (3–9)	7 (3–11)	0.59
**Type of disease**, ***n*** **(%)**			0.65
*TBI*	10 (40)	10 (36)	
*SAH*	14 (56)	15 (54)	
*ICH*	1 (4)	3 (10)	
*MED*	0	0	
**During ICU stay**			
Vasopressors, *n* (%)	25 (100)	28 (100)	–
Mechanical ventilation, *n* (%)	25 (100)	28 (100)	–
RRT, *n* (%)	1 (4)	0	0.47
Osmotic therapy, *n* (%)	21 (84)	24 (86)	1.00
Decompressive craniectomy, *n* (%)	7 (28)	3 (11)	0.16
Hypothermia, *n* (%)	4 (16)	6 (21)	0.73
Anti-epileptics, *n* (%)	24 (96)	24 (86)	0.36
Barbiturates, *n* (%)	12 (48)	9 (32)	0.27
Intracranial hypertension, *n* (%)	19 (76)	17 (61)	0.26
Seizures, *n* (%)	4 (16)	5 (18)	1.00
**On the first day of assessment**			
Body temperature, °C	37.0 (36.8–37.3)	36.9 (36.7–37.4)	0.69
PaCO_2_, mmHg	38 (35–39)	37 (36–38)	0.51
PaO_2_, mmHg	102 (98–110)	100 (98–111)	0.79
Lactate, mmol/L	1.1 (0.8–1.2)	1.0 (0.7–1.1)	0.12
Hemoglobin, g/dl	11.3 (10.8–12.2)	11.2 (10.7–12.2)	0.63
mFV, cm/sec	56.0 (49.7–71.0)	55.3 (47.3–62.7)	0.13
Pulsatility Index	0.91 (0.69–1.13)	0.92 (0.80–1.05)	0.41
Mean NPi	4.6 (4.3–4.7)	4.1 (3.2–4.6)	0.30
iPbtO_2_, mmHg	17 (16–19)	22 (20–26)	<0.01
Brain hypoxia, *n* (%)	20 (80)	6 (21)	<0.01
**Outcomes**			
ICU stay, days	15 (12–24)	23 (11–33)	0.18
Hospital mortality, *n* (%)	11 (44)	7 (25)	0.16
GOS 3 months	3 (1–4)	3 (1–4)	0.37
UO	16 (64)	17 (61)	1.00

*CKD, chronic kidney disease; COPD, chronic obstructive pulmonary disease; TBI, traumatic brain injury; SAH, subarachnoid hemorrhage; ICH, intracerebral hemorrhage; RRT, renal replacement therapy; PaCO_2_, partial arterial CO_2_ pressure; PaO_2_, partial arterial oxygen pressure; mFV-MCA, mean MCA flow velocity; NPI, neurological pupil index; iPbtO_2_, brain tissue oxygenation at baseline; ICU, intensive care unit; GOS, Glasgow Outcome Scale*.

**Table 4:** Changes in study variables on the first day of assessment, according to response to CPP challenge and baseline tissue hypoxia.

		**Step 1**	**Step 2**	**Step 3**	***p*-value**
Responders(*n* = 25)	MAP, mmHg	83 (80–89)	95 (90–98)	103 (100–108)	<0.01
	ICP, mmHg	11 (9–17)	11 (10–15)	11 (9–15)	0.26
	CPP, mmHg	72 (70–74)	82 (80–85)	91 (89–95)	<0.01
	PbtO_2_, mmHg	17 (16–19)	21 (18–22)	24 (22–26)	<0.01
No responders (*n* = 28)	MAP, mmHg	83 (82–88)	96 (92–99)	107 (101–110)	<0.01
	ICP, mmHg	12 (6–14)	12,5 (9–15)	12 (10–15)	0.01
	CPP, mmHg	74 (72–76)	84 (80–87)	94 (90–97)	<0.01
	PbtO_2_, mmHg	22 (20–26)	23 (22–26)	24 (22–27)	<0.01
Normal PbtO_2_(*n* = 27)	MAP, mmHg	83 (82–88)	95 (92–100)	105 (100–110)	<0.01
	ICP, mmHg	12 (6–14)	12 (9–14)	11 (9–14)	0.43
	CPP, mmHg	74 (71–76)	85 (81–88)	94 (91–97)	<0.01
	PbtO_2_, mmHg	23 (21–26)	24 (23–28)	26 (24–29)	<0.01
Tissue hypoxia(*n* = 26)	MAP, mmHg	86 (82–89)	95 (91–98)	106 (100–110)	<0.01
	ICP, mmHg	11 (9–17)	11 (10–17)	12 (9–18)	<0.01
	CPP, mmHg	72 (70–74)	82 (79–85)	91 (88–96)	<0.01
	PbtO_2_, mmHg	17 (16–18)	20 (18–22)	22 (21–24)	<0.01

*MAP, mean arterial pressure; ICP, intracranial pressure; CPP, cerebral perfusion pressure; PbtO_2_, Brain tissue oxygenation*.

**Figure 3:**
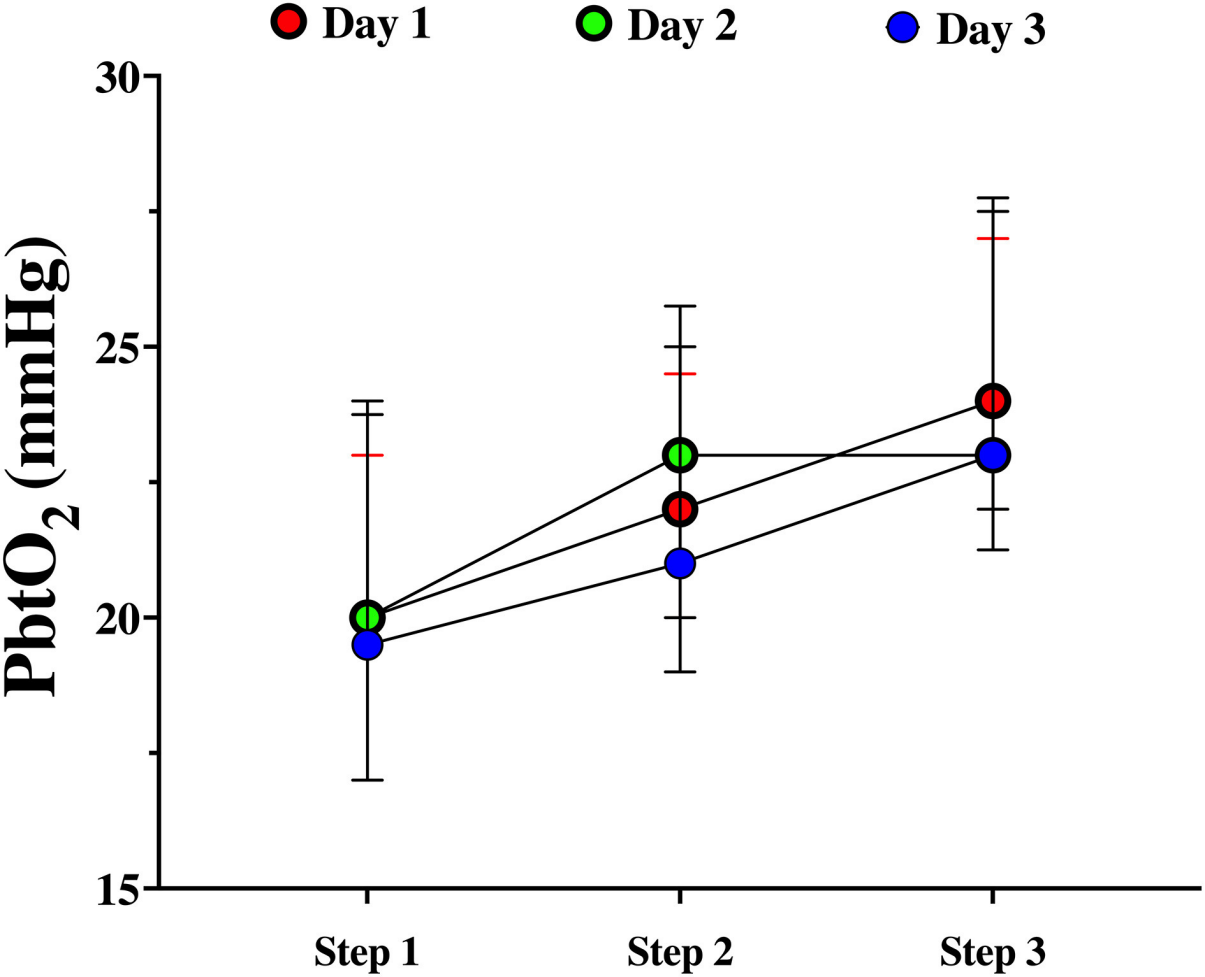
PbtO_2_ changes during the CPP challenge over the first 3 days of assessment.

Among patients with baseline tissue hypoxia on the first day of assessment (Supplementary Table 2), changes in PbtO_2_ were significantly higher in those with tissue hypoxia when compared to others (Table 4; Figure 2). Similar changes in PbtO_2_ between days 1, 2, and 3 were observed (Wald chi-square 6.42; *p* = 0.59; Figure 3); data on day 4 and 5 were not included into this analysis because of the high number of missing challenges.

A total of 19 (56%), 10 (42%), 4 (36%), and 3 (43%) were PbtO_2_ responders during the CPP challenge on days 2, 3, 4, and 5, respectively. A total of 18 (53%), 12 (50%), 4 (36%), and 3 (43%) had baseline brain tissue hypoxia at baseline on days 2, 3, 4, and 5, respectively.

A total of 123 paired assessment of PbtO_2_ with mFV and PI changes and 59 of PbtO_2_ with NPi changes were available over the first 5 days of assessment. No correlation of PbtO_2_ changes were observed with changes in mFV or PI (correlation index of −0.043 and −0.031, with *p*-values of 0.57 and 0.68, respectively), while a weak correlation between PbtO_2_ and NPi changes was observed (correlation index of 0.181, *p* = 0.017).

Differences between patients with UO and FO are shown in Table 1. Before the CPP challenge, baseline characteristics were similar between groups on day 1. Also, changes in PbtO_2_ over time on day 1 were not statistically significant between groups (Table 2; Figure 2).

## Discussion

In this study, including a heterogeneous population of brain injured patients, increasing CPP resulted in a significant increase of the brain oxygenation in most of patients. The “optimal” CPP, i.e., the CPP value corresponding to the absence of tissue hypoxia, was higher than in recommended targets (i.e., around 80 mmHg). Half of patients showed a significant increase in PbtO_2_ during the CPP challenge, in particular, if they had lower PbtO_2_ values at baseline. The effects of CPP on PbtO_2_ changes were similar in the following days of assessment. Non-invasive neuromonitoring could not adequately predict PbtO_2_ changes during CPP increase.

The improvement in PbtO_2_ values during CPP increase using vasopressors has also been reported in previous studies. Johnston et al. reported a significant increase in brain oxygenation when CPP was increased from 70 to 90 mmHg (8); these changes were also associated with an increase in cerebral blood flow and a decrease in oxygen extraction fraction (OER). However, as CPP challenge resulted in a greater proportional increase in PbtO_2_ than OER, the authors concluded that this intervention could potentially increase the oxygen gradient between the vascular and tissue compartments. In another study, increasing CPP also resulted into a significant increase in PbtO_2_ (9). Our study included a larger population of patients, included other diseases than TBI, and tested two different CPP targets above the baseline values. Importantly, this CPP challenge should be performed only in those patients with low PbtO_2_ values at the baseline, in the absence of intracranial hypertension or hypoxemia. Increasing CPP in patients with normal PbtO_2_ at baseline would result in less significant oxygen improvement and no theoretical effect on tissue metabolism. In one study, Stocchetti et al. also reported that PbtO_2_ regularly improved after 22 CPP challenges, in particular when low oxygen values were present at the baseline (16). Baseline PbtO_2_ below 20 mm Hg was often associated with CPP values within normal ranges. Interestingly, increasing CPP with other drugs than norepinephrine, such as dopamine, might result in less predictable CPP increase and less significant PbtO_2_ increase (17, 18). Moreover, PbtO_2_ would change in response to CPP challenge if placed into “at-risk” areas, as in our study, while effects might be minimal if the catheter is inserted into normal-appearing parenchyma (17).

It has been suggested that cerebral hemodynamics after an acute brain injury could be assessed using non-invasive monitoring, such as cerebral blood flow velocities or cerebral autoregulation indices. As such, because PbtO_2_ is dependent on CPP below the lower limit of autoregulation (i.e., the value of CPP corresponding to the direct dependency of cerebral blood flow from the systemic driving pressure) (19), recommended CPP targets can still result in tissue hypoxia in the absence of elevated ICP, as autoregulation might be impaired or the lower limit shifted toward higher CPP thresholds in brain injured patients (20). Individualized CPP values based on optimal autoregulation status has also been suggested in TBI patients (20); however, targeting “safe” CPP values based on the plateau curve of autoregulation could not correspond to adequate brain oxygenation in some patients (21). In one study (19), PbtO_2_ was pressure dependent when autoregulation was impaired, while it remained within stable values if autoregulation was intact. However, tissue hypoxia could occur even within normal autoregulation indices (19), thus suggesting that only the presence of PbtO_2_ monitoring could help to optimize CPP and avoid tissue hypoxia. As such, it is not surprising that changes in PbtO_2_ during the CPP challenge would not correlate with changes in mFV or PI, two parameters derived from the analysis of cerebral blood flow velocities that are commonly used to estimate cerebral hypoperfusion in the presence of intracranial hypertension (22). In SAH patients, no correlation was observed between PbtO_2_ measurements and, simultaneously, TCD recording (23). In another study including TBI patients, episode of cerebral hypoxia had all mFV <40 cm/s; however, no correlation was observed between PbtO_2_ and mFV in the whole cohort (24). Unfortunately, we did not specifically assess cerebral autoregulation in our study and could not provide additional data to the relationship between autoregulatory status and brain oxygenation. Similarly, NPi assessment using automated pupillometry could reflect elevated ICP (25); however, as tissue hypoxia may occur even within normal ICP values, the lack of sufficient correlation between PbtO_2_ and NPi changes was expected.

This study has several limitations to acknowledge. First, the small sample size and monocentric and retrospective design might introduce significant selection biases and limit the generalizability of our findings, although characteristics of included and excluded patients were similar. Second, although we suggested the need for higher than recommended CPP targets, MAP transducer was zeroed at the atrium level, while some centers would place the zero-reference point next to cerebral anatomical structures, i.e., the foramen of Monro, which would result in 10–15 mmHg lower CPP, depending on the elevation of the head of the bed (usually 15–30°) (26). Third, many patients had baseline ICP, CPP, and PbtO_2_ within normal values at baseline, which might not entirely justify a daily “CPP challenge.” However, the duration of CPP challenge was short, being supervised by an experienced intensivist, and was clinically relevant, as conducted in a well-designed approach, and informative for daily patient management. Interestingly, in those patients with normal PbtO_2_ levels at baseline, lower than recommended CPP targets might have been theoretically used, still maintaining adequate oxygen levels. However, lower CPP values would be outside of routine management in brain injured patients and future studies should prospectively evaluate the safety of such approach. Fourth, the procedure was considered as safe (i.e., no complications were recorded); however, we did not assess changes in cerebral metabolism (i.e., glucose or lactate/pyruvate ratio), which might have also been influenced by changes in brain hemodynamics. Fifth, CPP augmentation was not compared with other interventions able to increase PbtO_2_ in brain injured patients (i.e., increase in FiO_2_ and arterial hyperoxia) (27). As such, whether increased CPP rather than, as an example, increased FiO_2_ would result in similar effects on brain oxygenation or would have a different safety profile in brain injured patients remains unknown. Sixth, we only assessed short-terms effects of increasing CPP, while persistently high CPP values might be associated with an increased risk of acute respiratory failure, at least in TBI patients, and would not result in better neurological outcome than standard targets (28). Seventh, we defined tissue hypoxia as a PbtO_2_ <20 mmHg; ischemic thresholds for PbtO_2_ have been tested using the association of this value with patients' outcome; a recent study identified a threshold of 19 mmHg which adequately separated patients with unfavorable and favorable neurological outcome after TBI (29). However, the ischemic threshold might vary among different brain disease and within patients and the presence of concomitant metabolism monitoring (i.e., microdialysis) might be helpful to identify ischemic levels of PbtO_2_ when they would be associated with low cerebral glucose levels and increased lactate/pyruvate ratio. Eighth, the cerebrovascular pathophysiology among the different types of acute brain injury is different; in particular, SAH patients can develop delayed cerebral ischemia between 4 and 15 days after the injury, whereas cerebral hyperemia and intracranial hypertension may be more common in TBI patients. As such, our findings are also relevant to the very early phase after the injury and in the absence of uncontrolled intracranial hypertension. Finally, we provided different descriptive analyses without multivariable assessment, as the number of events was limited; however, multiple statistical comparisons exposes to the inflation of false positive tests within dependent datasets.

## Conclusions

In this heterogeneous population of acute brain injured patients, PbtO_2_ monitoring suggested the need for higher than recommended CPP targets to avoid tissue hypoxia. A higher response in brain oxygenation to the CPP challenge was observed in those patients with low PbtO_2_ values at baseline. The effects of CPP increase over brain oxygenation are consistent over time.

## Data Availability Statement

The raw data supporting the conclusions of this article will be made available by the authors, without undue reservation.

## Ethics Statement

The studies involving human participants were reviewed and approved by Ethical Committee of the Erasme Hospital. Written informed consent for participation was not required for this study in accordance with the national legislation and the institutional requirements.

## Author Contributions

FST, SS, and MK conceived the study protocol. AQ and FST performed the transcranial Doppler. MK, EG, and LP collected the data. HN, LP, and MK analyzed the data. FST, MK, JC, and OD drafted the present manuscript. SS, EG, LP, and AQ critically revised the manuscript. All authors read and approved the final manuscript.

## Conflict of Interest

FST is the Scientific Advisor for Neuroptics Inc. and received lecture fees from Integra LifeSciences. The remaining authors declare that the research was conducted in the absence of any commercial or financial relationships that could be construed as a potential conflict of interest.

## Publisher's Note

All claims expressed in this article are solely those of the authors and do not necessarily represent those of their affiliated organizations, or those of the publisher, the editors and the reviewers. Any product that may be evaluated in this article, or claim that may be made by its manufacturer, is not guaranteed or endorsed by the publisher.
