# Supplementary material for: Optimal Cerebral Perfusion Pressure Guided by Brain Oxygen Pressure Measurement
Source: Front Neurol. 2021 Oct 28;12:732830. doi: 10.3389/fneur.2021.732830 (PMC8581172; doi:10.3389/fneur.2021.732830)
Supplement: Supplementary file 1 [file Table_1.DOCX]

**Supplemental Material**

**Optimal cerebral perfusion pressure guided by brain oxygen pressure measurement**

*Matyas KOVACS^1^, Lorenzo PELUSO^1^, Hassane NJIMI^1^, Olivier DEWITTE^2^, Elisa BOGOSSIAN^1^, Armin QUISPE CORNEJO^1^_,_ Jacques CRETEUR^1^, Sophie SCHUIND^1^, Fabio Silvio TACCONE^1^*

*^1^Deparment of Intensive Care, Hopital Erasme*

*Université Libre de Bruxelles (ULB), Brussels, Belgium*

*^2^Deparment of Neurosurgery, Hopital Erasme*

*Université Libre de Bruxelles (ULB), Brussels, Belgium*

Corresponding author:

**Lorenzo PELUSO, MD**

Department of Intensive Care

Erasme Hospital

Université Libre de Bruxelles (ULB)

Route de Lennik, 808

1070 – Brussels (BELGIUM)

email: lorenzo.peluso@erasme.ulb.ac.be

**Supplemental Table 1.** Characteristics of included and excluded patients.

|  | **Included**  **(n=53)** | **Not included**  **(n=109)** | ***p value*** |
| --- | --- | --- | --- |
| ***DEMOGRAPHICS*** | | | |
| Age, years | 50 [40 – 58] | 52 [42 – 62] | 0.30 |
| Male Gender, n (%) | 31 (58) | 63 (58) | 1.00 |
| ***COMORBIDITIES*** | | | |
| Hypertension, n (%) | 14 (26) | 46 (42) | 0.58 |
| Heart Disease, n (%) | 2 (4) | 9 (8) | 0.51 |
| Alcohol, n (%) | 12 (23) | 25 (23) | 1.00 |
| Smoking, n (%) | 12 (23) | 25 (23) | 1.00 |
| Diabetes, n (%) | 7 (13) | 10 (9) | 0.43 |
| Previous neurological disease, n (%) | 2 (4) | 10 (9) | 0.34 |
| CKD, n (%) | 0 | 0 | - |
| COPD, n (%) | 4 (7) | 10 (9) | 1.00 |
| Immunosuppression, n (%) | 0 | 1 (1) | 1.00 |
| Cancer, n (%) | 3 (6) | 6 (5) | 1.00 |
| Liver Cirrhosis, n (%) | 1 (2) | 3 (3) | 1.00 |
| ***ON ADMISSION*** | | | |
| Glasgow Coma Scale | 7 [3 – 10] | 7 [3 – 11] | 0.63 |
| Type of disease, n (%) |  |  | 0.14 |
| *TBI* | 20 (38) | 35 (32) |  |
| *SAH* | 29 (55) | 50 (46) |  |
| *ICH* | 4 (7) | 22 (20) |  |
| *MED* | - | 2 (2) |  |
| ***DURING ICU STAY*** | | | |
| Vasopressors, n (%) | 53 (100) | 104 (95) | 0.17 |
| Mechanical Ventilation, n (%) | 53 (100) | 104 (95) | 0.17 |
| RRT, n (%) | 1 (2) | 1 (1) | 0.55 |
| Osmotic therapy, n (%) | 45 (85) | 96 (88) | 0.62 |
| Decompressive Craniectomy, n (%) | 10 (19) | 24 (22) | 0.69 |
| Hypothermia, n (%) | 10 (19) | 24 (22) | 0.69 |
| Anti-epileptics, n (%) | 48 (91) | 92 (84) | 0.34 |
| Barbiturates, n (%) | 21 (40) | 35 (32) | 0.38 |
| Intracranial hypertension, n (%) | 36 (68) | 67 (61) | 0.49 |
| Seizures, n (%) | 9 (17) | 19 (17) | 1.00 |
| ***OUTCOMES*** | | | |
| ICU stay | 21 [11 – 26] | 15 [6 – 22] | 0.02 |
| Hospital mortality, n (%) | 18 (34) | 58 (53) | 0.03 |
| GOS 3 months | 3 [1 – 4] | 1 [1 – 3] | 0.05 |
| UO | 33 (62) | 81 (76) | 0.10 |

CKD = chronic kidney disease; COPD = Chronic obstructive pulmonary disease; TBI = Traumatic brain injury; SAH = Subarachnoid hemorrhage; ICH = Intracerebral hemorrhage; RRT = Renal replacement therapy; ICU = Intensive care unit; GOS = Glasgow outcome scale; UO = unfavorable outcome

**Supplemental Table 2.** Characteristics of population according to baseline PbtO_2_ values on the first day of assessment (tissue hypoxia = PbtO_2_ < 20 mmHg).

|  | **Normal iPbtO_2_**  **(n = 27)** | **Tissue Hypoxia**  **(n = 26)** | ***P value*** |
| --- | --- | --- | --- |
| Age, years | 49 [37 – 59] | 52 [45 – 57] | 0.44 |
| Male Gender, n (%) | 17 (63) | 14 (54) | 0.58 |
| Hypertension, n (%) | 6 (22) | 8 (31) | 0.54 |
| Heart Disease, n (%) | 2 (7) | 0 | 0.49 |
| Alcohol, n (%) | 6 (22) | 6 (23) | 1.00 |
| Smoking, n (%) | 8 (30) | 4 (15) | 0.33 |
| Diabetes, n (%) | 5 (19) | 2 (8) | 0.42 |
| Previous neurological disease, n (%) | 2 (7) | 0 | 0.49 |
| CKD, n (%) | 0 | 0 | - |
| COPD, n (%) | 2 (7) | 2 (8) | 1.00 |
| Immunosuppression, n (%) | 0 | 0 | - |
| Cancer, n (%) | 0 | 3 (12) | 0.11 |
| Liver Cirrhosis, n (%) | 0 | 1 (4) | 0.49 |
| Glasgow Coma Scale | 7 [4 – 10] | 5 [3 – 11] | 0.25 |
| Type of disease, n (%) |  |  | 0.09 |
| *TBI* | 8 (30) | 12 (46) |  |
| *SAH* | 15 (55) | 14 (54) |  |
| *ICH* | 4 (15) | 0 |  |
| *MED* | 0 | 0 |  |
| Vasopressors, n (%) | 27 (100) | 26 (100) | - |
| Mechanical Ventilation, n (%) | 27 (100) | 26 (100) | - |
| RRT, n (%) | 0 | 1 (4) | 0.49 |
| Osmotic therapy, n (%) | 23 (85) | 22 (85) | 1.00 |
| Decompressive Craniectomy, n (%) | 4 (15) | 6 (23) | 0.50 |
| Hypothermia, n (%) | 5 (19) | 5 (19) | 1.00 |
| Anti-epileptics, n (%) | 24 (89) | 24 (92) | 1.00 |
| Barbiturates, n (%) | 9 (33) | 12 (46) | 0.41 |
| Intracranial hypertension, n (%) | 17 (63) | 19 (73) | 0.56 |
| Seizures, n (%) | 3 (11) | 6 (23) | 0.29 |
| Body temperature, °C | 37 [36.8 – 37.4] | 36.9 [36.7 – 37.3] | 0.74 |
| PaCO_2_, mmHg | 37 [36 – 39] | 37 [36 – 39] | 0.92 |
| PaO_2_, mmHg | 103 [98 – 111] | 100 [98 – 106] | 0.41 |
| Lactate, mmol/L | 0.9 [0.7 – 1.1] | 1.1 [0.8 – 1.2] | 0.11 |
| Hemoglobin, g/dL | 11.4 [10.8 – 12.2] | 11.3 [10.8 – 12.2] | 0.97 |
| mFV, cm/sec | 55.3 [47.5 – 63.7] | 55.7 [49.5 – 67.0] | 0.62 |
| Pulsatility Index | 0.91 [0.75 – 1.05] | 0.91 [0.71 – 1.08] | 0.90 |
| Mean NPi | 4.6 [4.1 – 4.6] | 4.6 [3.1 -4.7] | 0.82 |
| iPbtO_2_, mmHg | 23 [21 – 26] | 17 [16 – 18] | <0.01 |
| ICU stay | 22 [16 – 33] | 15 [10 – 24] | 0.08 |
| Hospital mortality, n (%) | 5 (19) | 13 (50) | 0.02 |
| GOS 3 months | 3 [3 – 4 ] | 1 [1 - 4] | 0.02 |
| UO | 15 (56) | 18 (70) | 0.40 |
| PbtO_2_-Responder | 5 (19) | 18 (70) | <0.01 |

CKD = chronic kidney disease; COPD = Chronic Obstructive Pulmonary Disease; RRT = Renal replacement therapy; ICU = Intensive care unit; GOS = Glasgow outcome scale; iPbtO2 = Brain tissue oxygenation at baseline; NPI = Neurological pupil index; mFV = mean MCA flow velocity; PaCO_2_ = Partial arterial CO2 pressure; PaO_2_ = Partial arterial oxygen pressure.
